# Tunable Spin and Orbital Torques in Cu-Based Magnetic Heterostructures

**DOI:** 10.1021/acs.nanolett.4c05170

**Published:** 2025-01-29

**Authors:** Silvia Damerio, Can O. Avci

**Affiliations:** Institut de Ciència de Materials de Barcelona, Campus de la UAB, Bellaterra 08193, Spain

**Keywords:** spin−orbit torque, orbital currents, solid-state gating, spin−orbitronics

## Abstract

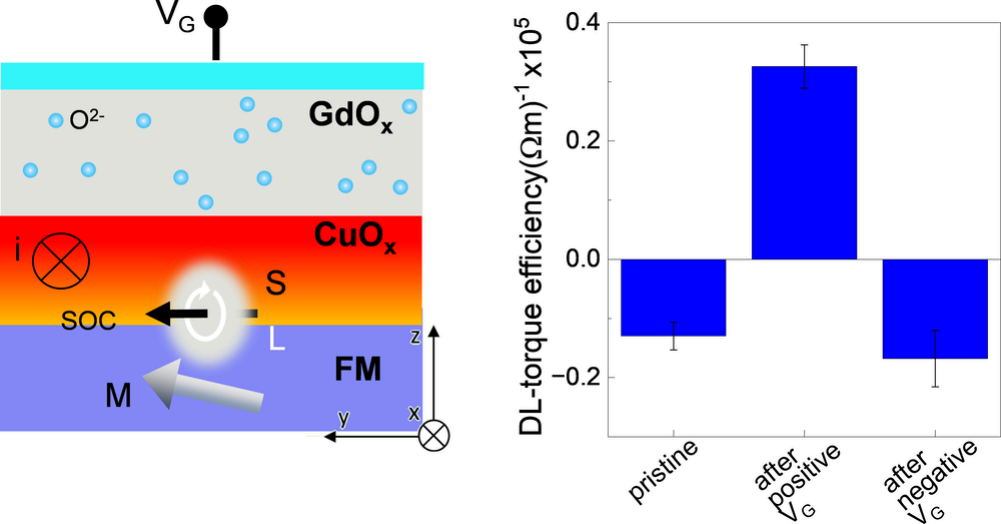

Current-induced torques originating from earth-abundant 3d elements offer a promising avenue for low-cost and sustainable spintronic memory and logic applications. Recently, orbital currents—transverse orbital angular momentum flow in response to an electric field—have been in the spotlight since they allow current-induced torque generation from 3d transition metals. Here, we report a comprehensive study of the current-induced spin and orbital torques in Cu-based magnetic heterostructures. We show that high torque efficiencies can be achieved in engineered Ni_80_Fe_20_/Cu bilayers where Cu is naturally oxidized, exceeding the ones found in the archetypical Co/Pt. Furthermore, we demonstrate sign and amplitude control of the damping-like torque by manipulating the oxidation state of Cu via solid-state gating. Our findings provide insights into the interplay between charge, spin, and orbital transport in Cu-based heterostructures and open the door to the development of gate-tunable spin–orbitronic devices.

The interconversion between charge and spin currents is a central topic in spintronics, allowing magnetization control through spin–orbit torques (SOTs).^1,2^ Traditionally, SOTs generation has mostly relied on the spin-Hall (SHE) and interfacial Rashba-Edelstein (REE) effects in materials characterized by strong spin–orbit coupling, thus limiting the platform choice to heavy metals such as Pt and W. Recently, the orbital Hall effect (OHE) has emerged as an alternative, involving the flow of orbital angular momentum perpendicular to a charge current.^3−5^ Unlike its spin counterpart, the OHE does not require strong spin–orbit coupling and thus is predicted to occur more broadly, with some 3d transition metals exhibiting greater orbital Hall conductivity than the best-known spin Hall materials.^6,7^ This has promoted efforts to demonstrate orbital currents, orbital momentum accumulation, and its conversion into orbital torques (OT) in ferromagnetic heterostructures.^8−15^

Cu is particularly intriguing in the context of spin–orbitronics. Previous studies show that the Cu/oxide interface can generate OTs,^16,17^ with efficiency enhancements achieved through natural oxidation,^18−23^ nitration,^24^ and interface engineering.^25,26^ Other effects such as unidirectional orbital magnetoresistance,^27^ orbital Hanle magnetoresistance,^28^ and orbital REE^11,29^ further highlight the intricate relationship between charge, orbital and spin degrees of freedom in Cu-based heterostructures. However, fundamental questions remain regarding the orbital texture needed for OHE in Cu and distinguishing between spin vs orbital and bulk vs interface contributions. Since orbital accumulation lacks direct interaction with magnetization through exchange coupling, orbital-to-spin conversion must occur at the interface or within the adjacent ferromagnet (FM),^30^ adding complexity to material selection and device design. Addressing these challenges is critical for developing efficient spin–orbitronic technologies.

In this study, we examined the (S)OTs induced in Co and Ni_80_Fe_20_ (Py) by adjacent Cu, Pt, and naturally oxidized CuO_*x*_ layers. In the structures with CuO_*x*_, we observe a damping-like OT with the opposite sign to that of the Pt and Cu references. Additionally, the FM thickness dependence with a fixed CuO_*x*_ layer demonstrates that the damping-like OT component increases significantly with increasing thickness of Co and Py. In particular, in Py/CuO_*x*_, the magnitude of OT efficiency becomes comparable to the SOT generated in the reference Py/Pt bilayers and exceeds that of Co/Pt when the Py thickness reaches 5 nm. A collective understanding of the data pinpoints the OHE as the origin of damping-like torque in FM/CuO_*x*_ structures. In addition, we demonstrate full electrical tunability of the damping-like OT in Co/CuO_*x*_/GdO_*x*_ solid-state gated devices by reversibly controlling the oxidation of the CuO_*x*_ layer through voltage-driven ion migration. Our findings support the potential of Cu to achieve efficient electrical control of magnetization, thereby encouraging further exploration of spin–orbitronic devices based on more economical and sustainable light elements.

Ti(1.5)/FM(*t_FM_*)/NM(3) (thicknesses in nm) with easy-plane magnetic anisotropy were deposited by d.c. magnetron sputtering onto SiO_*x*_ substrates (methods in Supporting Information Note 1). Here, Ti was used to improve adhesion and does not play an active role in the device functionality. The FMs are Co or Py, whereas the NMs are Cu or Pt. We either capped the final structure with 1.5 rm of Ti or left it uncapped to obtain unoxidized and partially oxidized Cu, respectively (see Supporting Information Note 2). *t*_*FM*_ was varied between 1.5 and 10 nm for each FM/NM combination. The saturation magnetization (*M*_*s*_) of Co and Py was found between 1.17–1.25 MA/m for Co depending on *t*_*Co*_, and 0.68 MA/m for Py, independently of *t*_*Py*_ (see Supporting Information Note 3). These values agree with previous reports^12,19^ and indicate a good quality of the deposited FM films. To quantify the (S)OTs, we patterned the heterostructures in Hall bars, depicted in Figure 1a, and performed harmonic Hall measurements.^31,32^

**Figure 1:**
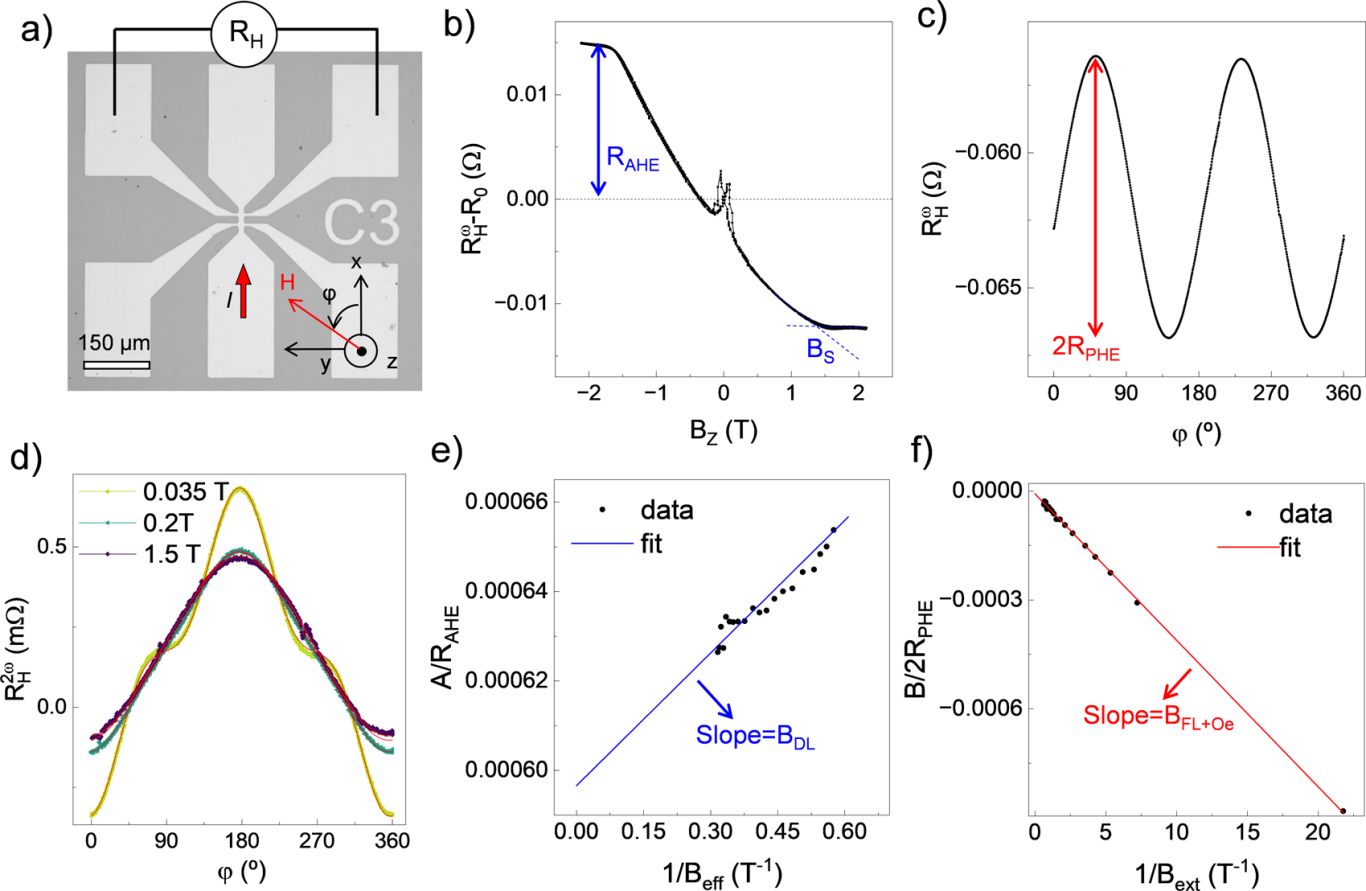
SOT quantification by harmonic Hall measurements. (a) Optical microscopy image of a representative Hall bar device and measurement scheme. (b) First harmonic Hall resistance (*R*_*H*_^ω^) as a function of out-of-plane field (*B*_*Z*_). (c) *R*_*H*_^ω^ as a function of in-plane angle (φ) measured at 1T. (d) Second harmonic Hall resistance (*R*_*H*_^2ω^) as a function φ measured at three representative external fields from 0.035 to 1.5 T. (e) Plot of the normalized cos φ component of *R*_*H*_^2ω^(φ) as a function of inverse effective field (1/*B*_*eff*_) and (f) plot of the normalized 2 cos^3^ φ – cos φ component of *R*_*H*_^2ω^(φ) as a function of inverse external field (1/*B*_*ext*_).

First, we extracted the anomalous Hall resistance (*R*_*AHE*_) and the saturation field (*B*_*S*_) by measuring the first harmonic Hall resistance (*R*_*H*_^ω^) during a swept out-of-plane field (*B*_*Z*_) as shown in Figure 1b. Next, we measured *R*_*H*_^ω^ and the second-harmonic Hall resistance (*R*_*H*_^2ω^) as a function of in-plane angle (φ) with constant external field (*B*_*ext*_). The former provides the planar Hall resistance (*R*_*PHE*_), as shown in Figure 1c, while the latter, plotted in Figure 1d for 3 representative fields, contains the contribution of SOTs, and thermoelectric contributions, as follows:^30,32,33^
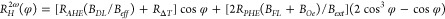
1Here *B*_*DL*_ and *B*_*FL*_ are the damping-like and field-like SOT effective fields, *B*_*Oe*_ is the current-induced Oersted field,^34,35^*B*_*eff*_ is the effective magnetic field calculated as *B*_*eff*_ = *B*_*ext*_ + *B*_*S*_, and *R*_Δ*T*_ represents the thermoelectric contributions, predominantly from the anomalous Nernst and spin Seebeck effects due to an out-of-plane temperature gradient.^32^ After quantifying the individual torque components as detailed in Supporting Information Note 1, the torque efficiency is calculated taking into account *M*_*S*_ and *t*_*FM*_ and normalized by the applied electric field (*E*):
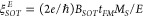
2

(S)OTs for Co(*t*)/NM(3) bilayers are summarized in Figure 2. Figure 2a shows *B*_*DL*_ values normalized by *E* as a function of *t*_*Co*_ in Co(*t*)/CuO_*x*_ (red penthagon), Co(*t*)/Cu (orange triangle) and Co(*t*)/Pt (gray circle). In a typical SOT system such as Co/Pt where *B*_*DL*_ is predominantly driven by the SHE, *B*_*DL*_ is expected to decrease with the increasing *t*_*Co*_,^22^ as is the case in our Co/Pt data. However, in Co/CuO_*x*_ and Co/Cu *B*_*DL*_ increases with *t*_*Co*_ instead. Here, *B*_*DL*_ is more than an order of magnitude lower than those generated from Pt, indicating generally low efficiency of using Cu as the torque generator acting upon Co. However, when naturally oxidized CuO_*x*_ is used, *B*_*DL*_ has the opposite (negative) sign compared to the Co/Cu and reference Co/Pt bilayers, but the same sign as the inverted Pt/Co (squares in Figure 2a,b). This intriguing result will be elaborated on later. Figure 2b shows the corresponding damping-like (S)OT efficiency (ξ_*DL*_^*E*^) calculated following eq 2. For Co/Pt, the magnitude of ξ_*DL*_^*E*^ is in line with previous reports^1^ and remains essentially constant (gray line in Figure 2b) with a minor increase for *t*_*Co*_ > 5 nm. On the other hand, ξ_*DL*_^*E*^ increases monotonically in Co/CuO_*x*_ and Co/Cu as a function of *t*_*Co*_ (note that the values are multiplied by 4 for better readability), indicating an enhanced efficiency with an increased magnetic volume, contrary to the standard behavior of SOTs.

**Figure 2:**
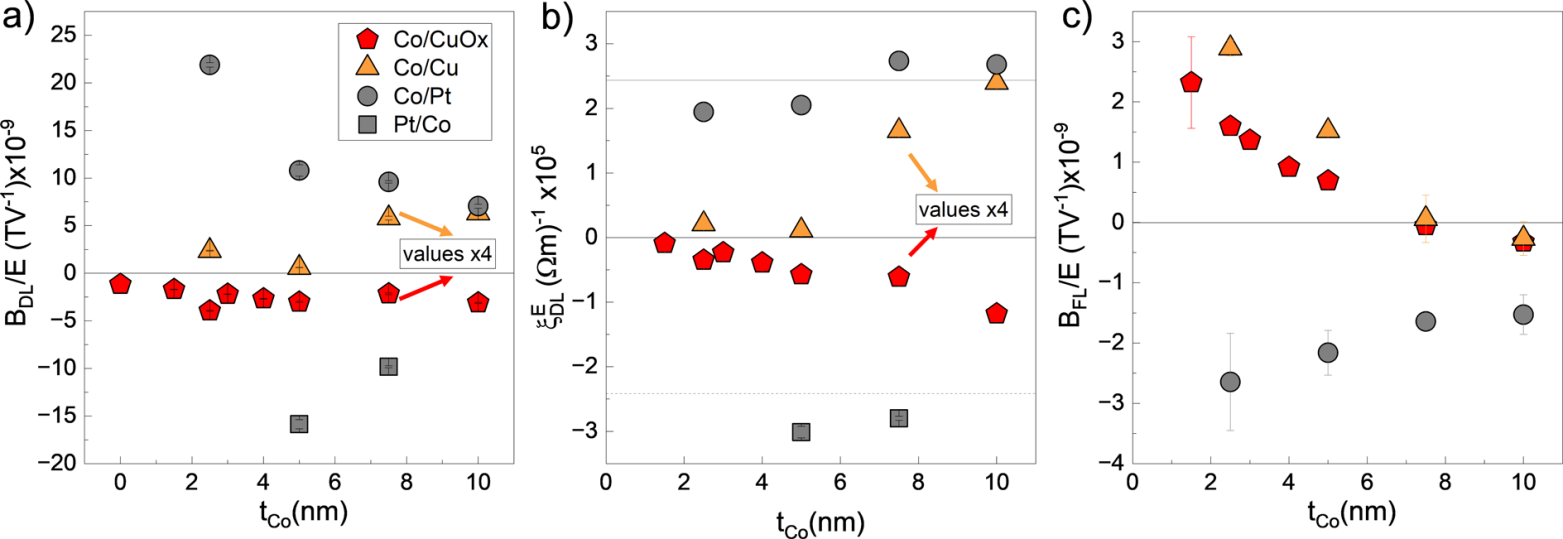
(S)OTs in Co(*t*)/NM(3) bilayers. Plot of (a) the damping-like torque normalized effective field (*B*_*DL*_/*E*), (b) the damping-like torque efficiency (ξ_*DL*_^*E*^), and (c) the field-like torque normalized effective field (*B*_*FL*_/*E*) as a function of Co thickness (*t*_*Co*_) for NM = CuO_*x*_ (red pentagon), NM = Cu (orange triangle), and NM = Pt (gray circle and square).

Now, we turn to the field-like (S)OT in Co(*t*)/NM(3) structures. Figure 2c shows *B*_*FL*_ as a function of *t*_*Co*_ after subtracting the *B*_*Oe*_ contribution (see Supporting Information Note 4). In Co/Pt, *B*_*FL*_ is negative and decreases in magnitude with the increasing *t*_*Co*_, following approximately the same trend as *B*_*DL*_, pinpointing their common origin. On the other hand, *B*_*FL*_ is positive in both Co/Cu and Co/CuO_*x*_ with comparable magnitude to those obtained in Co/Pt. Both data sets show a similar decreasing trend as a function of *t*_*Co*_, contrasting with the *B*_*DL*_ trends observed in these systems. This observation indicates a different origin of *B*_*FL*_ than that responsible for *B*_*DL*_ in Co/Cu and Co/CuO_*x*_. We tentatively attribute the source of *B*_*FL*_ to the Co/Cu interface, common in these two systems, where mechanisms like the interfacial REE could be at play. Overall, the SOTs quantification in Figure 2 suggests that Cu, CuO_*x*_, and their interfaces with Co are potential sources of (S)OTs. The opposite sign of *B*_*DL*_ in Cu vs CuO_*x*_ and their *t*_*Co*_ dependence provide strong evidence of an unconventional torque component that could be attributed to orbital angular momentum accumulation in CuO_*x*_ that is then converted to spin-torque in Co.

Next, we measured (S)OTs in equivalent Py(*t*)/NM structures to test the above hypothesis. Here, replacing Co with Py allows higher OHE contribution on the torques, given the larger spin–orbit conversion coefficient of Py.^9,36^ The (S)OT results for Py(*t*)/NM bilayers are summarized in Figure 3. Similarly to Co-based bilayers, here *B*_*DL*_ also scales with 1/*t*_*Py*_ when Pt is used as torque generator (gray circles in Figure 3a), and changes sign (but not in amplitude) when the stack is inverted (gray squares), establishing the SHE as the main source for *B*_*DL*_. In contrast, in Py/CuO_*x*_ (red pentagon in Figure 3a), *B*_*DL*_ increases as a function of *t*_*Py*_ up to 5 nm and then follows a decreasing trend. Similar behavior was observed in CoFe/CuO_*x*_^22^ and associated with the orbital origin of the spin accumulation in CuO_*x*_. For the orbital accumulation to generate torque on the magnetization, orbital-to-spin conversion needs to occur in the first few nm of the FM layer, leading to an initial sharp increase of *B*_*DL*_ with *t*_*FM*_ followed by an exponential decrease as the process saturates. Finally, in Py/Cu (orange triangle in Figure 3a), a very low and positive SOT is observed.

**Figure 3:**
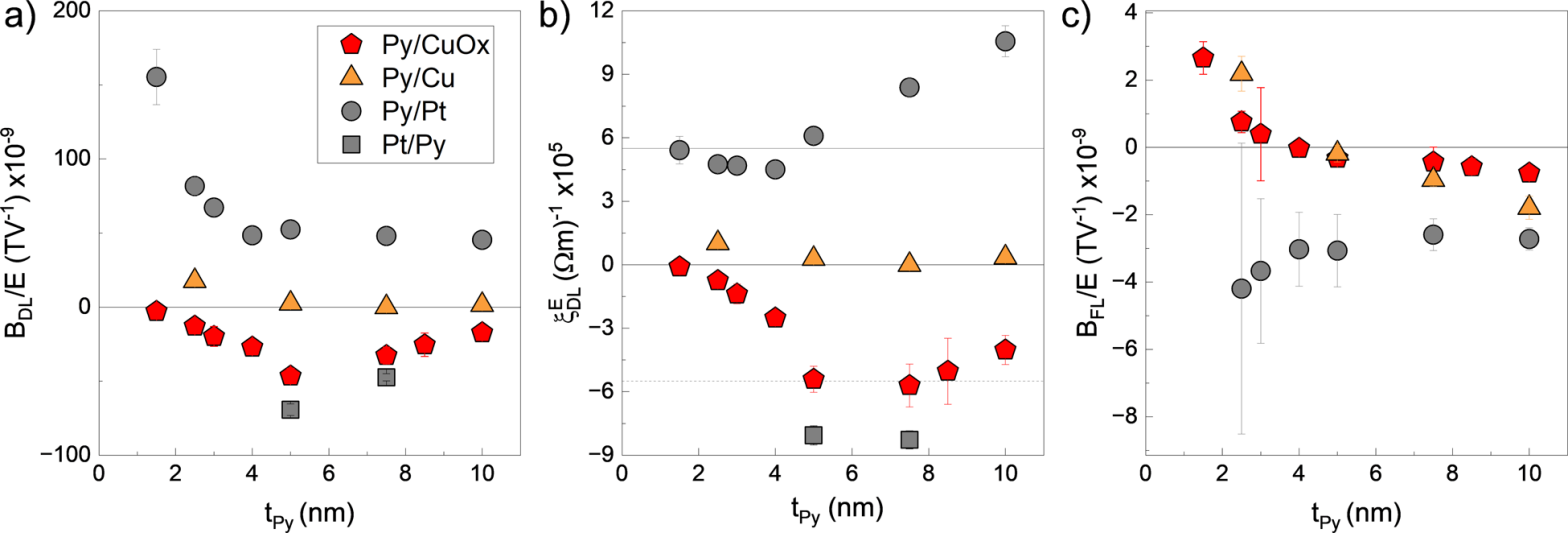
SOTs in Py(t)/NM(3) bilayers. Plot of (a) the damping-like torque normalized effective field (*B*_*DL*_/*E*), (b) the damping-like torque efficiency (ξ_*DL*_^*E*^), and (c) the field-like torque normalized effective field (*B*_*FL*_/*E*) as a function of Py thickness (*t*_*Py*_) for NM = CuO_*x*_ (red pentagon), NM = Cu (orange triangle), and NM = Pt (gray circle and square).

Figure 3b shows ξ_*DL*_^*E*^ for all Py(*t*)/NM structures with a highly contrasting outcome to that found in Co(*t*)/NM(3) reported in Figure 2b. First, ξ_*DL*_^*E*^ in Py/CuO_*x*_ is significantly larger than in Co/Pt, Co/CuO_*x*_ and Py/Cu, approaching the efficiency found in Py/Pt (gray dashed line). Second, ξ_*DL*_^*E*^ of Py/Pt is constant up to 5 nm with magnitudes up to three times larger than that found in Co/Pt and increases further for larger *t*_*Py*_. The significantly enhanced SOT efficiency compared to Co/Pt, together with the increasing trend for larger Py thickness, could indicate OHE contribution in Pt, which is reported to have the same sign as the SHE.^7^ Another possibility is the self-induced torque in Py.^37^ However, we characterized this in single Co and Py layers, and *B*_*DL*_ was found to be negligible in both cases (see Supporting Information Note 5). Conversely, with CuO_*x*_, ξ_*DL*_^*E*^ sharply increases up to 5 nm and then tends to saturate to a value comparable to ξ_*DL*_^*E*^ of Pt, but with the opposite sign (dotted gray line in Figure 3b). As mentioned earlier, this trend corroborates with the expected behavior of orbital currents, which need a certain amount of magnetic thickness to be fully converted into a spin current and hence exert torque on the magnetization. This thickness is characterized as the orbital diffusion length and depends on the FM choice. Assuming that *B*_*DL*_ is predominantly driven by orbital currents, in the case of Py, the orbital diffusion length is around 5 nm, whereas in Co, it seems to be >10 nm (see Supporting Information Note 6). However, further experiments are required to validate these numbers and assumptions.

Finally, the *t*_*Py*_ dependence of *B*_*FL*_ is displayed in Figure 3c. Here, contrary to the situation with *B*_*DL*_, *B*_*FL*_ shows similarity to the case of Co(*t*)/NM. *B*_*FL*_ in Py/Pt is slightly larger than in Co/Pt, although the error bar is also large. *B*_*FL*_ in Py/Cu and Py/CuO_*x*_ shows the same qualitative trend of Co-based structures but undergoes a sign reversal and saturation toward a small negative value. We do not have a plausible explanation for the sign crossover and non-negligible *B*_*FL*_ at large Py thicknesses in Cu and CuO_*x*_ samples except the possible underestimation of the *B*_*Oe*_ contribution, which would erroneously lead to the reported trend.

The large difference of ξ_*DL*_^*E*^ between Co/CuO_*x*_ and Py/CuO_*x*_ can be explained by considering the spin–orbit conversion mechanism at the FM/CuO_*x*_ interfaces. In FM/NM bilayers, the overall charge-to-spin conversion efficiency (θ_*SH*_) is given by the sum of spin (σ_*SH*_^*NM*^) and orbital (σ_*OH*_^*NM*^) conductivity normalized by the electrical conductivity (σ^*NM*^):^9^
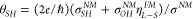
3

As apparent from eq 3, the orbital contribution to θ_*SH*_ depends on the spin–orbit conversion coefficient (η_*L*–*S*_^*FM*^) of the ferromagnet. While for Py, due to its high Ni content, we expect a large spin–orbit conversion coefficient, this term is much smaller in Co (η_*L*–*S*_^*Ni*^>η_*L*–*S*_^*Co*^).^36^ Hence, the orbital moment accumulation generated due to the OHE in CuO_*x*_ is more efficiently converted to a torque on the magnetization of Py than Co. Moreover, when Cu is not oxidized, we observe a small positive amplitude of the damping-like torque in Co/Cu bilayers and almost no torque in Py/Cu. This is in agreement with theoretical calculations that predict both σ_*SH*_^*NM*^ and σ_*OH*_^*NM*^ to be small and positive in metallic Cu.^8^ It is plausible to assume that in the Py/Cu case, small reminiscent oxidation counteracts the spin hall effect, making the total torque even smaller than in the Co/Cu case. Nevertheless, we find that natural oxidation of Cu is a viable method to increase the generation of orbital currents in this light metal.

The negative sign of ξ_*DL*_^*E*^ in the FM/CuO_*x*_ bilayers of this study and its dependence on *t*_*FM*_ together point toward an orbital origin of the spin accumulation. Negative-sign torque on Co has also been observed before in Co/CuN_*x*_ by Chen et al.,^24^ and its sign had been attributed to a negative L-S conversion coefficient in Co (η_*L*–*S*_^*Co*^), whereas An et al. reported positive orbital torques in Py/CuO_*x*_.^16^ On the other hand, Xiao et al.^19^ found a significant enhancement of the OT efficiency in Pt/Co/Cu–CuO_*x*_ heterostructures, where the OT derived from orbital angular momentum accumulation in Cu-CuO_*x*_ and the SOT provided by the Pt layer drive the rotation of the magnetization collaboratively. Considered the geometry of the stack, this means that the OT from Cu-CuO_*x*_ and the SOT from Pt on Co have an opposite sign, which corresponds to our observations. These discrepancies denote that the mechanism for the orbital angular momentum accumulation is highly dependent on the structural property and thickness of CuO_*x*_ and its interface with FMs. We postulate that different oxidation conditions can lead to different orbital textures that influence the sign of the torque. Moreover, the interplay between bulk and interface-based processes, namely the OHE and the orbital REE, can also vary based on differences in the interface quality (roughness, intermixing, etc.) and whether or not the Cu layer is fully oxidized or a Cu-CuO_*x*_ interface is still present. It is also worth mentioning that ξ_*DL*_^*E*^ of the reference Py/Pt is about twice that of Co/Pt. This cannot be attributed to a difference in the quality of the Pt growth on different FMs, because the same magnitude of torque is obtained when the stacking sequence of the layers is reversed (square dots in Figure 2a-b and Figure 3a-b). Therefore, this indicates an intrinsic difference in the spin transparency of the Py/NM and Co/NM interfaces, favoring the former for both spin and orbital torque applications. The critical role of the spin transparency at the FM/NM interface is also evident when utilizing Ni as the FM layer. We observe comparable torques between Ni/CuO_*x*_ and Ni/Pt, as expected of the large orbital-to-spin conversion efficiency of Ni. However, the overall (S)OTs efficiency is about 2 orders of magnitude lower with respect to the Py-based systems (see Supporting Information Note 7) due presumably to the low spin/orbital transmission at the Ni/NM interface.

The sign change of ξ_*DL*_^*E*^ between Co/Cu and Co/CuO_*x*_ bilayers, arising from turning on and off the OHE in Cu by changing its orbital texture, can be exploited to tune the OT via full-electric control of the oxidation state of Cu. To demonstrate this, we fabricated solid-state gated devices, where voltage-driven O^2–^ ion migration across a GdO_*x*_ insulating barrier can controllably and reversibly oxidize Cu. This approach is readily used in magnetoionics to modulate magnetic^38^ and interface properties of thin films^39,40^ and in engineering SOTs via oxygen manipulation.^41,42^ However, electrical control of orbital currents has so far only been shown indirectly via measurement of the spin Hall magnetoresistance and through the use of liquid ion gating,^20^ which is incompatible with implementation in microelectronics. Therefore, our experiments offer a more direct and scalable approach to technological applications.

Figure 4a shows a micrograph of a representative device overlaid with simplified electrical connections. We used GdO_*x*_(25 nm) as the dielectric barrier and Pt(5 nm) as the top gate (Figure 4b). We note that Co(5)/CuO_*x*_(3) was prepared using the same protocol as the devices measured earlier, hence Cu is naturally oxidized. When a positive gate voltage (+*V*_*G*_) is applied across the GdO_*x*_, we expect negatively charged O^2–^ ions to be attracted toward the top Pt contact (Figure 4b),^43^ deoxidizing CuO_*x*_. –*V*_*G*_ will then drive the *O*^2–^ ions from GdO_*x*_ into the Cu layer and oxidize it.

**Figure 4:**
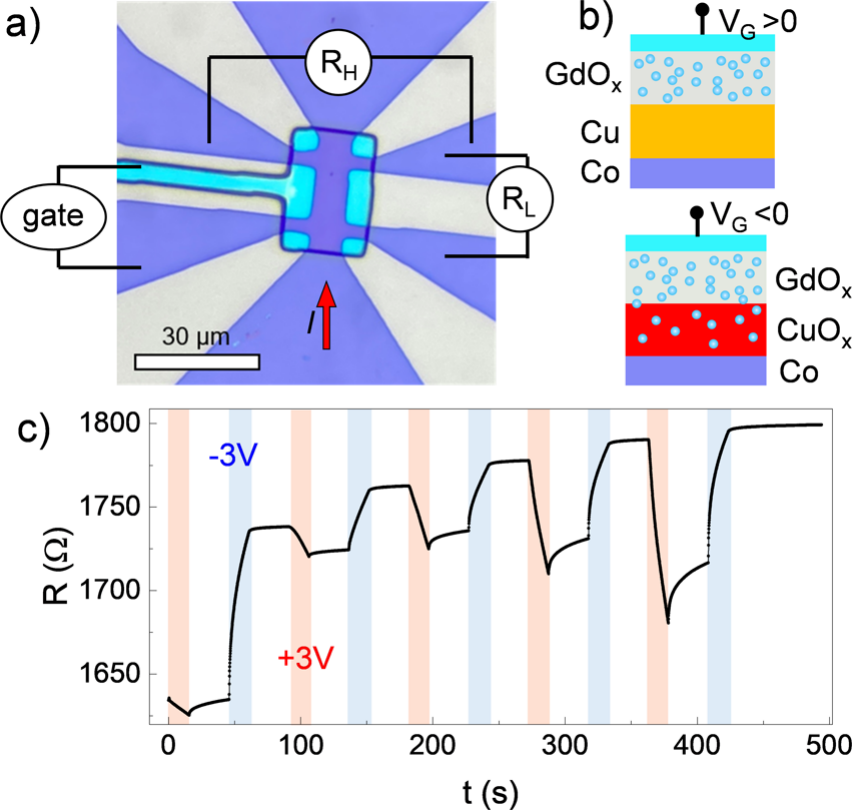
Solid-state gated devices. (a) Optical microscope image of the device and schematics of the connections. (b) Schematic representation of the voltage-driven *O*^2–^ ions (blue spheres) migration across the cross-section of the sample. (c) Plot of longitudinal resistance *R*_*L*_ as a function of time during application of a positive (red), negative (blue) or zero (white) gate voltage.

The voltage-driven O^2–^ migration is reflected in a resistance change as (part of the) Cu loses or regains its metallic character. Figure 4c displays the evolution of the resistance (*R*) measured in the 2-point geometry of a prototypical device when gated with *V*_*G*_ = ±3 V. +*V*_*G*_ (red) decreases *R*, while −*V*_*G*_ (blue) increases it. The relative change in *R* increases after each gate iteration, suggesting a training effect owing to the ion mobility increasing after several gating cycles. Moreover, when *V*_*G*_ is not applied (white), *R* tends to increase slightly and then saturate, especially after +*V*_*G*_. This can be explained by an initial partial reduction of Gd and its natural tendency to reoxidize once *V*_*G*_ is removed. On the other hand, once Gd gets reoxidized, *R* remains essentially constant over the experimental time frame (see Supporting Information Note 8), indicating that voltage-driven ion migration is an effective means to the stable reduction of CuO_*x*_ to metallic Cu.

Figure 5 shows the OT efficiency (defined in eq 2) measured at the three stages of the gating cycle for two different devices. In these experiments, *V*_*G*_ is applied following the sequence 0 V → + 3 V → 0 V → −3 V → 0 V. In both cases, ξ_*DL*_^*E*^ is negative in the pristine state, confirming that the CuO_*x*_ layer is initially oxidized (details of the fabrication process in Supporting Information Note 1). In Device 1, *V*_*G*_ = +3 V is applied for an arbitrarily long time of 750s, during which *R* decreases ≈3.5%. After + *V*_*G*_, the torque sign becomes positive, as expected when the spin accumulation originates from metallic Cu. It is worth mentioning that upon negative gating a Cu/GdO_*x*_ is created which could exhibit the orbital REE, thereby producing an additional positive component to the torque.^26^ Subsequently, we apply −*V*_*G*_ for 1250s, after which *R* increases ≈5% compared to its initial value in the pristine state. Measuring the OTs after negative gating reveals that the sign of the damping-like torque is reversed back to negative due to the oxidation of Cu.

**Figure 5:**
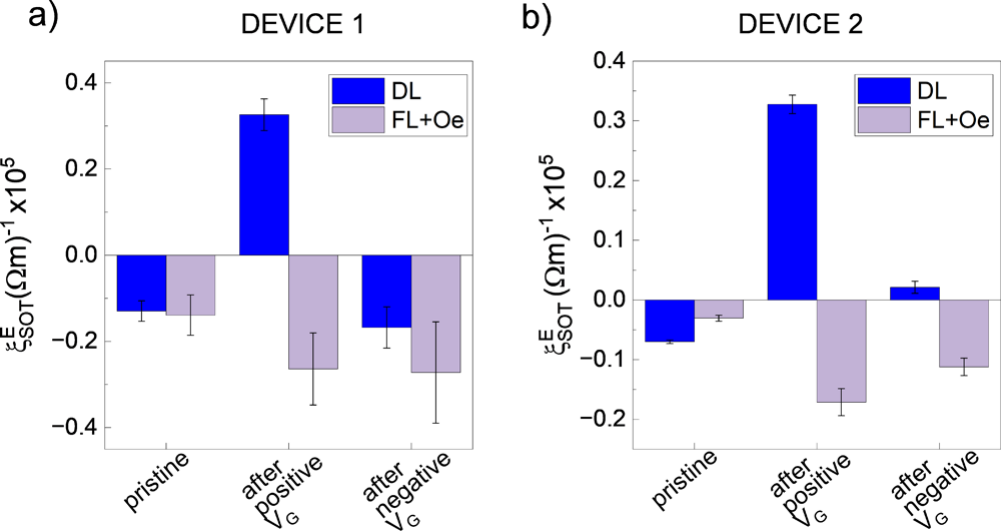
Electrical control of orbital torques. Plot of the damping-like (ξ_*DL*_^*E*^) (blue) and field-like + Oersted (ξ_*FL* + *Oe*_^*E*^) (violet) torque efficiency measured before gating (pristine) and after application of a positive and negative gate voltage (*V*_*G*_). Gate cycle (a) with and (b) without sign reversal of the damping-like torque efficiency.

Varying the duration of *V*_*G*_ allows us to tune the oxidation state of Cu continuously and, hence, the magnitude of the damping-like torque as exemplified in Device 2. Applying +*V*_*G*_ for 1000s followed by −*V*_*G*_ for 2000s leads to an almost complete suppression of the net torque. The *R*_*H*_ vs *H*_*Z*_ loops at the various stages of the experiment have also been recorded. Here, we observe that the amplitude of the AHE changes, owning to the change in current density across the FM, but *B*_*S*_ remains essentially unvaried. Because *B*_*S*_ is directly proportional to *M*_*S*_, it can be used as an indication of potential oxidation of the FM layer. In our experiments, we conclude that a small unintentional oxidation of Co, if any, does not affect the amplitude or sign of the torque (see Supporting Information Note 8).^44,45^

The field-like OT, differently from the damping-like component, remains negative across the gating sequence, as expected in the case of 5 nm thick Co (see Figure 2c). However, its magnitude increases as the resistance decreases, owing to the larger current in the Cu layer and, thus, larger Oersted contribution. This further confirms the different origins of *B*_*FL*_ and *B*_*DL*_ in Co/Cu and Co/CuO_*x*_ structures.

We have repeated similar gating experiments in multiple devices over multiple samples and verified that the general behavior is reproducible. However, we also observed large variability in the exact values of ξ_*DL*_^*E*^. We attribute these differences to the inhomogeneity of the natural oxidation of Cu that is also reflected in differences in the device resistance and gating times required for the oxidation/reduction of Cu. Further optimization is needed before this concept can be implemented for future application. Overall, our experiments demonstrate the feasibility of voltage-driven ion migration to control the oxidation state of Cu. Despite long gating times limited by the slow O^2–^ diffusion, Figure 5 is a clear proof of concept for the possibility of dynamical control of OTs in spin–orbitronic devices.

In conclusion, we have studied the (S)OTs in Co(*t*_*Co*_)/NM and Py(*t*_*Py*_)/NM bilayers (NM = Py, Cu, CuO_*x*_) utilizing harmonic Hall effect measurements. Comparing the results between different NM and analyzing the FM thickness dependence of the (S)OTs, we conclude that the damping-like component predominantly originates from the SHE in Pt and the OHE in CuO_*x*_. Owning to the large orbital-to-spin conversion coefficient in Py, the OT in Py/CuO_*x*_ is comparable in magnitude to the reference Py/Pt bilayer and exceeds that found in Co/Pt. This confirms that the OHE in this material is large and can be harnessed in spin–orbitronics devices. Moreover, the damping-like torque efficiency changes sign based on the oxidation state of Cu. We exploit this to demonstrate reversible tuning of the OT in Co(5)/CuO_*x*_(3) via voltage-driven ion migration in solid-state gated devices. Our study is a major step toward harnessing orbital currents in cost-effective light elements to achieve efficient and dynamic electrical control of magnetization in spin–orbitronics devices.

## Supporting Information Available

Methods, XPS analysis, VSM measurements, parallel resistor model of current density, self-induced torques, spin and orbital diffusion length, spin transparency of the FM/NM interface, and voltage-driven ion migration in Cu (PDF)
