# Supplementary material for: Tunable Spin and Orbital Torques in Cu-Based Magnetic Heterostructures
Source: Nano Lett. 2025 Jan 29;25(6):2181–7. doi: 10.1021/acs.nanolett.4c05170 (PMC11827597; doi:10.1021/acs.nanolett.4c05170)
Supplement: Supplementary file 1 — nl4c05170_si_001.pdf [file nl4c05170_si_001.pdf]

Supporting Information for "Tunable spin and orbital torques in Cu-based magnetic heterostructures"

Silvia Damerio[1,*] and Can O. Avci[1]

[1]*Institut de Ciència de Materials de Barcelona, Campus de la UAB, Bellaterra, 08193, Spain*

*sdamerio@icmab.es

Table of contents

1-Methods……………………………………………………………………………1
2-XPS analysis…………………………………………………………………………3
3-VSM measurements…………………………………………………………………4
4-Parallel resistor model of current density……………………………………………5
5-Self-induced torques…………………………………………………………………6
6-Spin and orbital diffusion length ……………………………………………………8
7-Spin transparency of the FM/NM interface……………………………………………9
8-Voltage-driven ion migration in Cu …………………………………………………10

1. Methods

<u>Sample Preparation.</u> Bilayers of Co/Cu, Co/Pt, Py/Cu and Py/Pt with in-plane magnetic anisotropy were deposited on thermally oxidized Si(001)/SiO2 substrates at room temperature by DC magnetron sputtering with 3 mTorr Ar. The deposition rates of Co, Py, Cu and Pt were 0.104 nms^{-1} at 200 W, 0.0568 nms^{-1} at 100 W, 0.0675 nm nms^{-1} at 50 W and 0.185 nms^{-1} 1 at 50 W, respectively. Adhesion and capping (where specified) Ti layers were also deposited at 200 W with a rate of 0.057 nms^{-1}. To obtain Co/CuO_X and Py/CuO_X, the fabricated bilayers were left in air overnight to naturally oxidize Cu before the transport measurements. For the gated devices, after lift-off of the patterned metallic stack, a continuous layer of GdO_X was deposited by RF magnetron sputtering with 3 mTorr Ar:O_2 (30:5) at 50 W and deposition rate of 0.0064 nms^{-1}. Finally, Pt contacts and the top gate were patterned and deposited. The resistance through the GdO_X spacer was > 100MΩ ensuring negligible crosstalk.

<u>Magnetic Measurements.</u> The magnetic properties of the continuous layers were measured using vibrating sample magnetometry in a Quantum Design Physical Property Measurement System (PPMS).

<u>Transport measurements.</u> For the transport measurements, the Co/CuO_X and Py/CuO_X films were patterned into six-terminal Hall bar devices by standard laser-

writer optical lithography and lift-off. The Hall bar dimensions are 30 μm for the current line length, 7.5 μm its width, and 3 μm the Hall branch width. The anomalous Hall voltage and angular-dependent first- and second-harmonic voltages were measured using a Zurich Instruments MFLI digital lock-in amplifier under an ac current $I = I_0 sin(\omega t)$ of amplitude I_0 = 6 − 12 mA and frequency ω = 2.9 kHz provided by a Keithley 6221 current source. For the gating experiments, a dc voltage of ±3V was applied between the top gate and one of the Hall arms with a Keithley 2470 Source Meter. All measurements were performed at room temperature.

SOT quantification. The damping-like and field-like (S)OT contributions can be separated based on their angular dependence from the fit of the second-harmonic Hall resistance $R^{2\omega}_H (\varphi)$ with a function of the type $A cos\varphi + B(2cos^3\varphi - cos\varphi)$ (red in Figure 1d of the main text). The damping-like (B_{DL}) and fiel-like effective fields (B_{FL}) are thus obtained from the slope of the normalized $cos\varphi$ and $2cos^3\varphi - cos\varphi$ components as a function of $1/B_{eff}$ and $1/B_{ext}$, respectively (Figure 1e-f of the main text):

$$A/R_{AHE} = B_{DL}/B_{eff}$$

$$B/2R_{PHE} = (B_{FL} + B_{Oe})/B_{ext}$$

Note that the B_{Oe} contribution can be estimated by the current in the NM layer (I_{NM}) using a parallel resistor model (see Note 4) and following the formula $B_{Oe} = \mu_0 I_{NM}/2*w$, where w =7.5 μm is the width of the Hall bar.

X-Ray diffraction. The polycrystalline structure of Py films has been investigated by means of grazing incidence X-ray diffraction (GIXRD) in a Bruker D8-Discover diffractometer with Cu K-alpha radiation (λ =1.5406 Å). In the spectrum, shown in Figure S1, we observed a single peak at 2θ=44.3º which agrees with the (111) diffraction peak of face-centered cubic (FCC) $Ni_{0.8}Fe_{0.2}$ with lattice parameter 3.55 Å [*A. V. Svalov et al., Sensors 22(21), 8357 (2022)*], indicating that the films are polycrystalline and do not deviate significantly from the nominal target stoichiometry (Fe 20% Ni 80%).

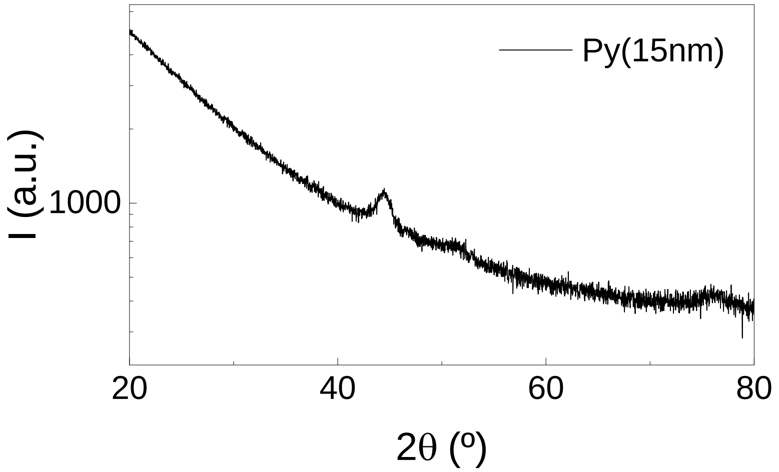

Figure S1 Py crystal structure. GIXRD pattern of a 15 nm thick Py film grown on Si substrate with a 1.5 nm Ti buffer layer.

2. XPS analysis

To characterize the composition of our materials, the oxidation state of Ti, O, Cu and Co was investigated by X-ray Photoelectron spectroscopy (XPS), as shown in Figure S2.

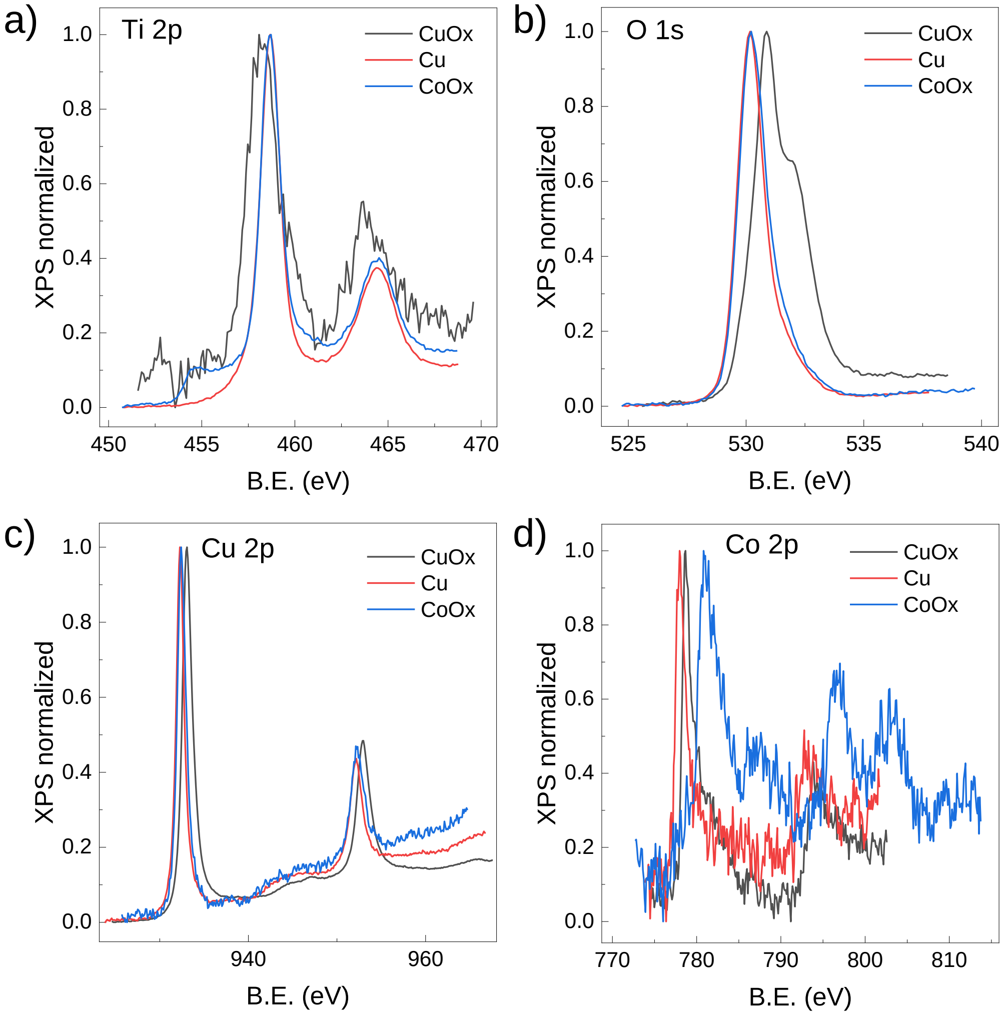

Figure S2 XPS spectra. a) Ti 2p, b) O 1s, c) Cu 2p and d) Co 2p regions of the XPS spectrum of a Ti(1.5)/Co(5)/CuO_X(3) (gray), Ti(1.5)/Co(5)/Cu(3)/Ti(1.5) (red) and a Ti(1.5)/CoO_X(6)/Cu(3)/Ti(1.5) (blue) samples.

We prepared three different samples: 1- Ti(1.5)/Co(5)/CuO_X (3), where CuO_X was obtained by natural oxidation of Cu, 2 - Ti(1.5)/Co(5)/Cu(3)/Ti(1.5) and 3- Ti(1.5)/CoO_X(6)/Cu/Ti(1.5), where CuO_X was obtained by natural oxidation of Co. The spectra were collected on a SPECS PHOIBOS 150 system and analyzed with the software CasaXPS. As it can be seen from the Ti 2p region (Figure S2a) the samples with Ti as top capping layer (red and blue) display a strong TiO_2 2p peaks, while the noisier peaks in the oxidized specimen (gray) originate from the bottom Ti

layer used for adhesion. The O 1s region of the Ti capped samples (Figure S2b) displays a single peak, indicating that all the Ti is uniformly oxidized. On the other hand, the presence of multiple peaks in the oxidized sample indicates that the oxidation of the top Cu layer is not uniform across the sample thickness. This is also reflected in the Cu region of the spectrum (Figure S2c), where the Cu 2p peaks of the oxidized sample is shifted towards higher binding energies compared to the other two samples. Finally, looking at the Co 2p region (Figure S2d), a significant difference is only found in peaks of the sample where Co was exposed to air, indicating that in the other two the oxidation doesn't reach Co.

3. VSM measurements

The magnetic properties of the FM(*t*)/Cu(3) bilayers were studied by means of vibrating sample magnetometry in a Quantum Design Physical Property Measurement System (PPMS). Figure S3a shows the in-plane magnetic hysteresis loops of Co samples with variable thickness. The saturation magnetization M_S of Co ranges from 1.17×10^6 A/m for the 1.5 nm thick film to 1.25×10^6 A/m for 5 nm thick one. This reduction of M_S compared to the bulk value of 1.44×10^6 A/m [*J.M.D. Coey, "Magnetism and Magnetic Materials" Cambridge University Press (2010)*], is not uncommon in thin films and is usually attributed to reduced dimensionality, microstructural variations and interface effects. Previous works *[C. O. Avci et al. Physical Review B 100, 235454 (2019); S. Lee et al.*, *Communications Physics 4, 234 (2021)]* report similar values for Co films below 10 nm and show that it also depends on the underlying layer. Conversely, the M_S of Py (Figure S3b) doesn't vary significantly with thickness and is 6.8×10^5 A/m for both 2.5 and 10 nm thick samples, close to bulk value.

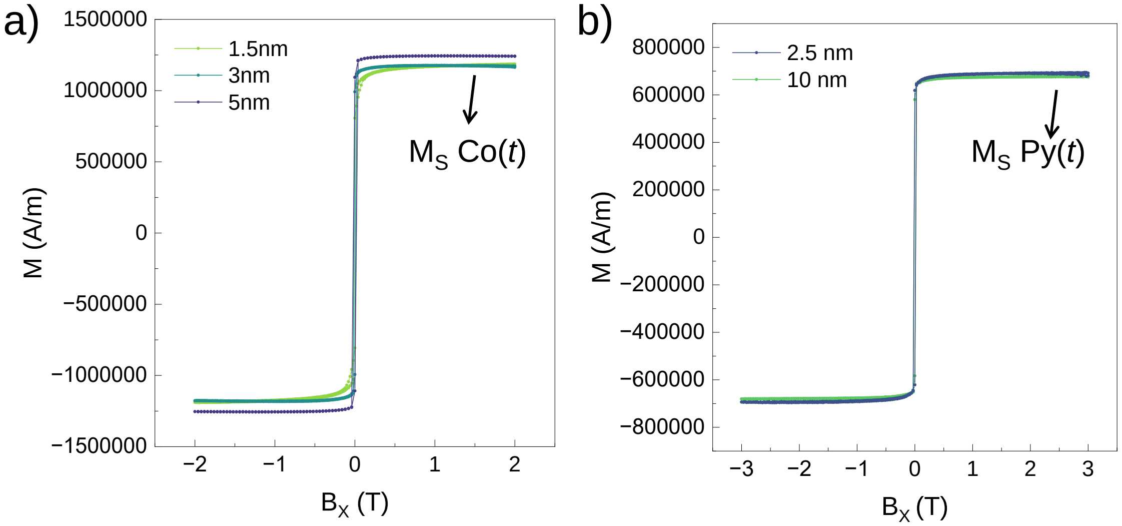

Figure S3 M-H loops. a) Plot of magnetization (*M*) as a function of in-plane field (B_X) of Co films with thickness of 1.5, 3 and 5 nm. b) Plot of *M* as a function of B_X of Py films with thickness of 2.5 and 10 nm.

4. Parallel resistor model of current density

The resistivity of Co, Py, Cu and Pt are measured from reference single-layer samples and result to be 37.2 μΩ/cm, 51.8 μΩ/cm, 8.8 μΩ/cm and 50 μΩ/cm respectively. These values are comparable with what previously obtained for films of comparable thickness [*Y. Ke, et al., Phys. Rev. B 79, 155406 (2009), Nguyen M-H. et al., Phys. Rev. Lett. 116, 126601 (2016)*]. Similar resistivity values for Co and Py can also be obtained from the fit of the FM layer thickness dependence of the inverse sheet resistance ($1/R_S$) of the samples, as shown in Figure S4a and Figure S4b. The resistivity of the individual layers is then used to calculate the current density in the NM, which is given by:

$$j_{NM} = \frac{i}{wt_{NM}} \frac{t_{NM}\rho_{FM}}{t_{NM}\rho_{FM} + t_{FM}\rho_{NM}}$$

where *i* is the total current injected in the device and *w*=7.5 μm is the width of the Hall bar.

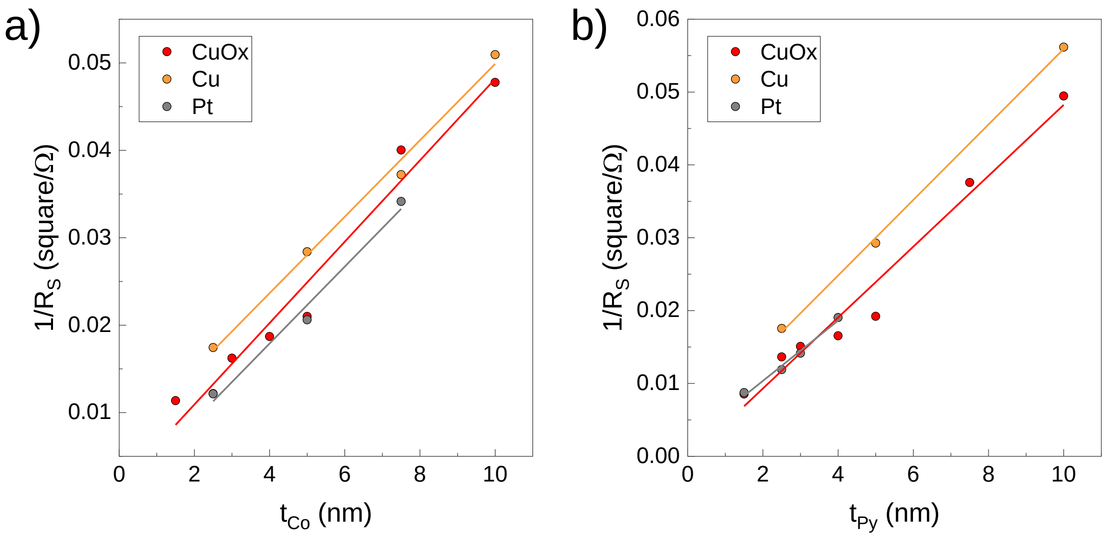

Figure S4 Resistance of Co and Py. Plot of the Inverse sheet resistance ($1/R_S$) as a function of a) Co thickness (t_{Co}) and b) Py thickness (t_{Py}) in Co(t)/NM(3) and Py(t)/NM(3) bilayers with NM=CuO_x (red), NM=Cu (orange) and NM=Pt (gray).

The Oersted filed generated by the current flowing in the NM can be then calculated as using:

$$B_{Oe} = -\frac{\mu_0 i_{NM}}{2w}$$

where μ_0 is the vacuum permeability and w the width of the Hall bar device. We used the values of B_{Oe} obtained in this way to estimate B_{FL} in Figures 2c and 3c of the main text.

Figure S5 shows the plot of the effective field of the field-like and Oersted component of the torque measured experimentally (open symbols) and the calculated B_{Oe} (green lines) as a function of thickness of the FM. As it can be seen, the amplitude of B_{Oe} decreases as expected with increasing t_{FM}, because the current in the NM decreases. The same trend is observed for B_{FL+Oe} for FM/Pt bilayers. However, when CuO_X is used as torque generator the trend reverses. This indicates the presence of a field-like component of the torque with opposite sign with respect to that generated in Pt that competes with the Oersted component.

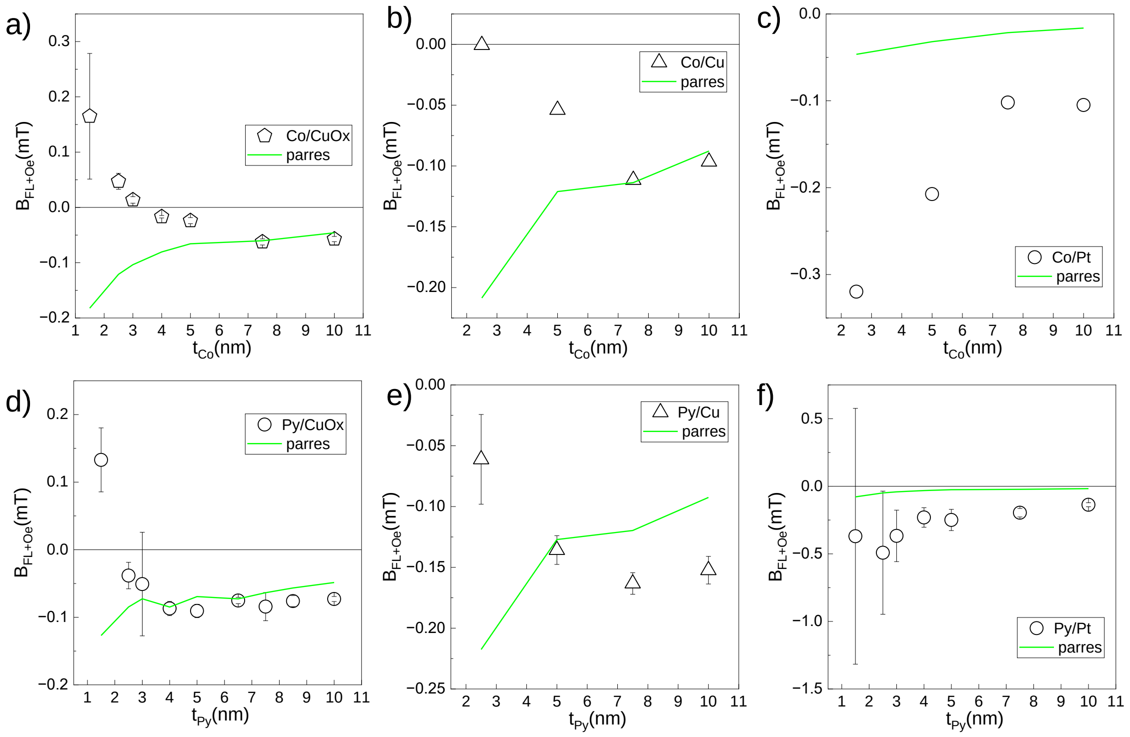

Figure S5 Current induced Oersted field. Plot of the field-like and Oersted effective field (B_{FL+Oe}) as a function of Co thickess (t_{Co}) for a) Co(t)/CuO_X(3), b) Co(t)/Cu(3) and c) Co(t)/Pt(3) bilayers. Plot of B_{FL+Oe} as a function of Py thickess (t_{Py}) for d) Py(t)/CuO_X(3), e) Py(t)/Cu(3) and f) Py(t)/Pt(3) bilayers. The green lines represent the calculated B_{Oe}.

5. Self-induced torques

Spin accumulations can also be generated by spin-polarized currents in metallic ferromagnets without the active intervention of adjacent normal metals [*H. Ochoa, R. Zarzuela, and Y. Tserkovnyak, J. Magn. Magn. Mater. 538, 168262 (2021)*]. To estimate the magnitude of the self-induced torque in Co and Py we measured single-layer films. Figure S6a-b show the comparison of the torque on a 5 nm Co film with

different capping layers. As apparent, both damping-like and field-like component of the torque are close to zero in the absence of an adjacent NM (self-torque). Small positive damping-like torque is observed for Cu capping layer, whereas CuOx provides a negative torque. A similar negative torque is observed when the Co sample is exposed to air before the deposition of the Cu(3)/Ti(1.5). This indicates that the formation of CoOx at the interface enhances the SOT efficiency, as observed in Co/CoOx/Pt [*X. Feng, et al., Appl. Phys. Lett. 118, 132410 (2021)*]. The same is found for Py based bilayers (Figure S6c-d): no self-torque is observed without capping layer, but, differently from the Co case, we also found no significant enhancement of the SOT upon oxidation of Py.

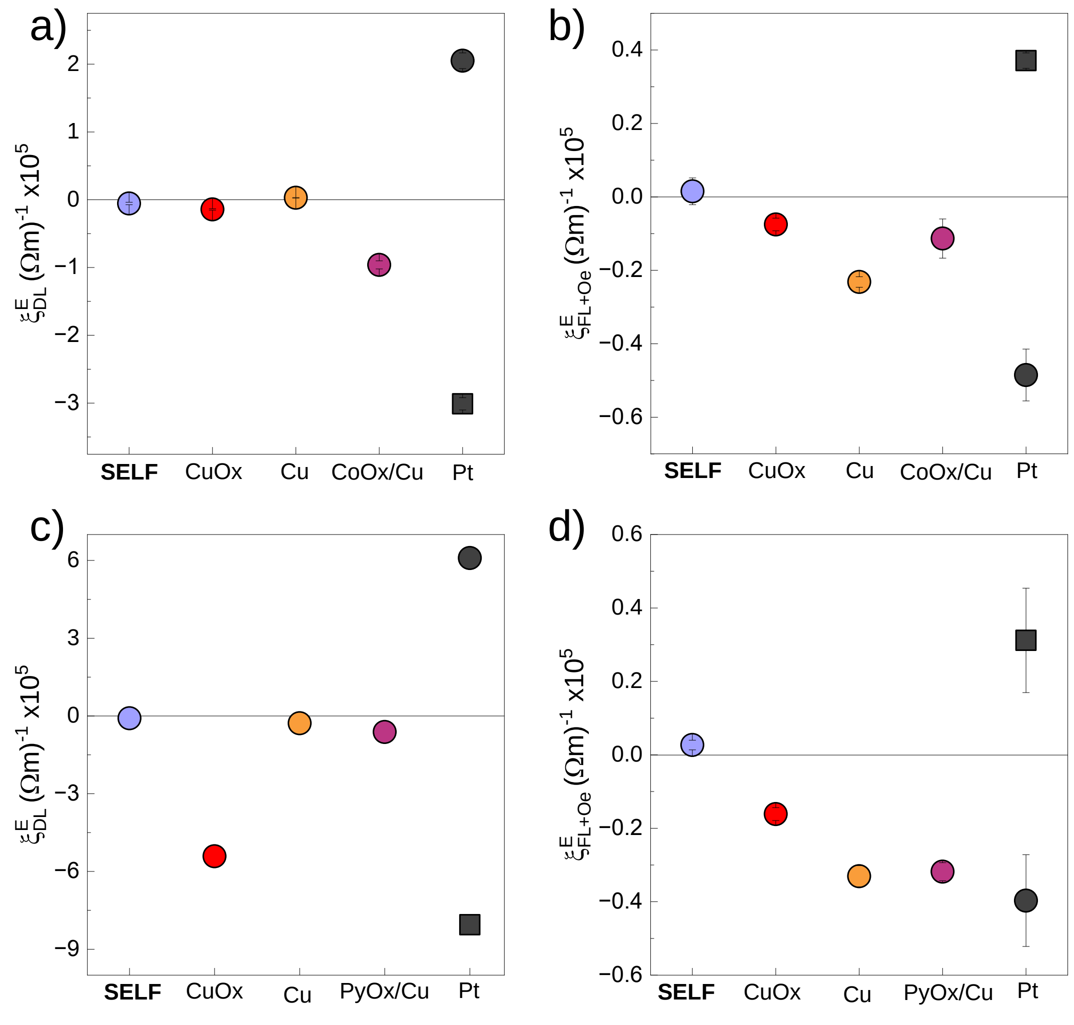

Figure S6 Comparison of capping layer on SOTs. a) Damping-like torque efficiency (ξ^E_{DL}) and b) field-like and Oersted torque efficiency (ξ^E_{FL+Oe}) in different Co(5)/NM bilayers. c) ξ^E_{DL} and d) ξ^E_{FL+Oe} in different Py(5)/NM bilayers.

6. Spin and orbital diffusion length

To quantify the spin diffusion length in Pt (λ_{Pt}) we studied the SOT in Co(5)/Pt(*t*) bilayers. Figure S7a shows a plot of the damping-like component of the torque as a function of Pt thickness (t_{Pt}), which increases steeply for the first few nm and saturates above 3 nm. From the fit of the experimental points with a hyperbolic tangent function (green line) we estimate λ_{Pt} in our system to be 1.56 nm, in good agreement with previous reports [*S. Ding et al., Nano Letters 24 (33), 10251–10257 (2024)*]. Figure S7b also shows the field-like and Oersted component of the torque as a function of Pt thickness (t_{Pt}). Here we see that the experimental points lie above the estimated Oersted component (black), thus indicating a positive field-like torque.

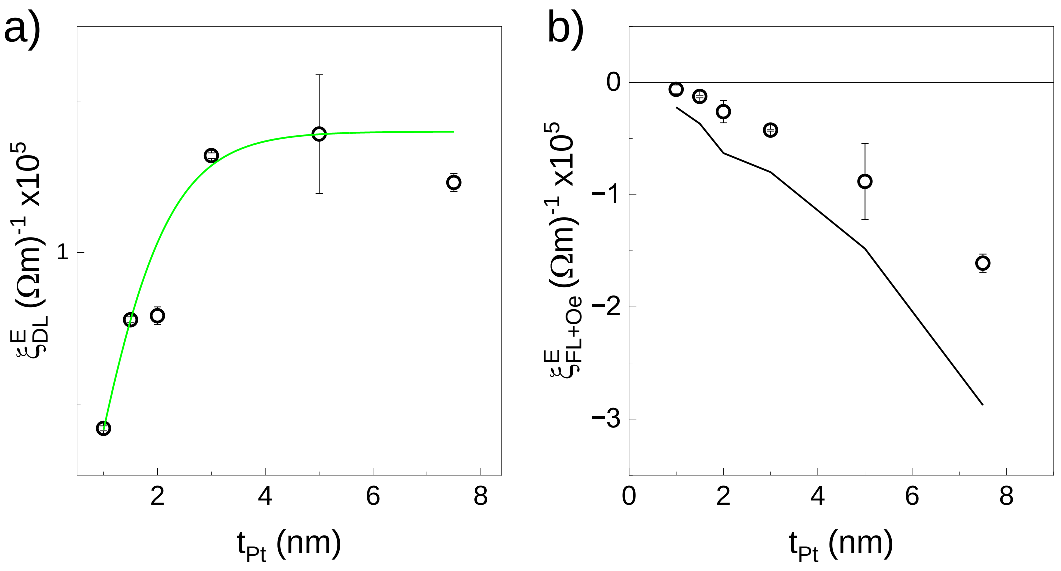

Figure S7 Spin diffusion length in Co(5)/Pt(t) bilayers. a) Damping-like torque efficiency (ξ^E_{DL}) and b) field-like and Oersted torque efficiency (ξ^E_{FL+Oe}) as a function of Pt thickness (t_{Pt}). The green solid line indicates the fit with a hyperbolic tangent function, while the black solid line indicates the estimated Oersted field generated by the current flow.

We have also made an attempt to estimate the saturation value of the orbital diffusion length in FM/CuO_X(3) bilayers. To do so, we have fitted the data in Figure 3b of the manuscript using an hyperbolic tangent function (green in Figure S8a) of the type: *y=A*tanh(x/B+C)+D,* where λ_{Py}=*B+C.* Setting C=1.5 nm, the smallest value of t_{Py} that we measured we obtain λ_{Py}≈4 nm. This value is somewhat lower than what found by Hayashi and coworkers *[H. Hayashi et al. Communication Physics 6,32 (2023)*] who have seen that the damping-like torque efficiency increases with t_{FM} up to t_{Ni} > 20 nm in Ni/Ti bilayers, but still larger than the spin diffusion length in Pt. We have also tentatively repeated the same fit for the data in Figure 2b of the manuscript and extract λ_{Co}, however the results of the fit (shown in Figure S8b) are in this case less reliable as we don't see any saturation of ξ_{DL} up to the largest thickness. Therefore, in the case of Co we can only conclude that λ_{Co}≈4.5 nm only represents a lower limit to the actual orbital diffusion length

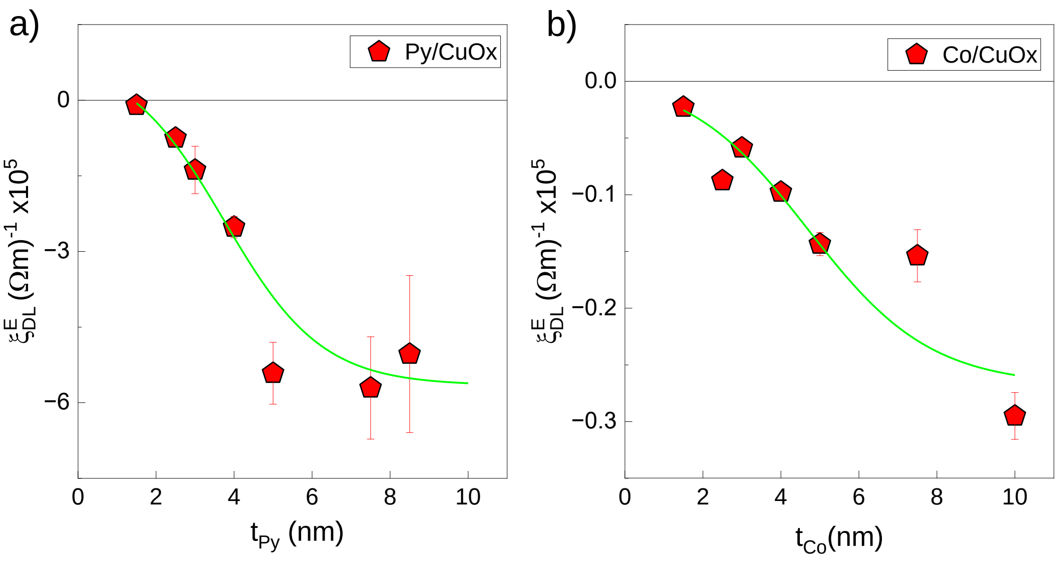

Figure S8 Orbital diffusion length in FM/CuOx(3) bilayers. a) Damping-like torque efficiency (ξ^{E}_{DL}) of a) Py thickness (t_{Py}). and b) Co thickness (t_{Co}). The green solid line indicates the fit with a hyperbolic tangent function.

7. Spin transparency at the FM/NM interface

It is well-known that the bulk of Ni is more efficient than Py in the orbital-to-spin conversion. Therefore, we attempted to measure the orbital torques in Ni/CuOx bilayers. Figure S9a shows the damping-like torque efficiency (ξ_{DL}) measured in different FM/NM bilayers. Surprisingly, the absolute value of ξ_{DL} for Ni(5)/CuOx(3) (blue) is two orders of magnitude lower than in the Py/CuOx(3) case (green). However, because the same amplitude difference is found when comparing the two reference samples Ni(5)/Pt(3) and Py(5)/Pt(3), we conclude that the origin of the lower signal is related to the interfacial spin/orbital injection efficiency at the Ni/NM interfaces presumably due to more diffuse interface, making this material less suitable for harmonic Hall measurements. On the other hand, using Co as a FM, ξ_{DL} with Pt is one order of magnitude larger than with CuOx (red in Figure S9a). The collective data corroborate our thesis that that Py and Ni are more efficient than Co for converting orbital currents that originate from CuOx. For a better comparison of the three FM metals, Figure S9b shows a plot of the ratio between ξ_{DL} measured in each FM/CuOx and corresponding FM/Pt reference. In view of the much larger amplitude of the signal, choosing Py over Ni is more sensible for the study of the FM thickness dependence of ξ_{DL}. Furthermore, we also noticed that the sign of ξ_{DL} for Ni(5)/CuOx(3) is positive. We hypothesize that this is related to the sign of the orbital-to-spin conversion in Ni, that could be opposite than that of Co and Py, in the same way as the AHE is also opposite in sign in Ni. Because both quantities depend on the interplay of their electronic band structure, Berry curvature, and spin-orbit coupling they could be related.

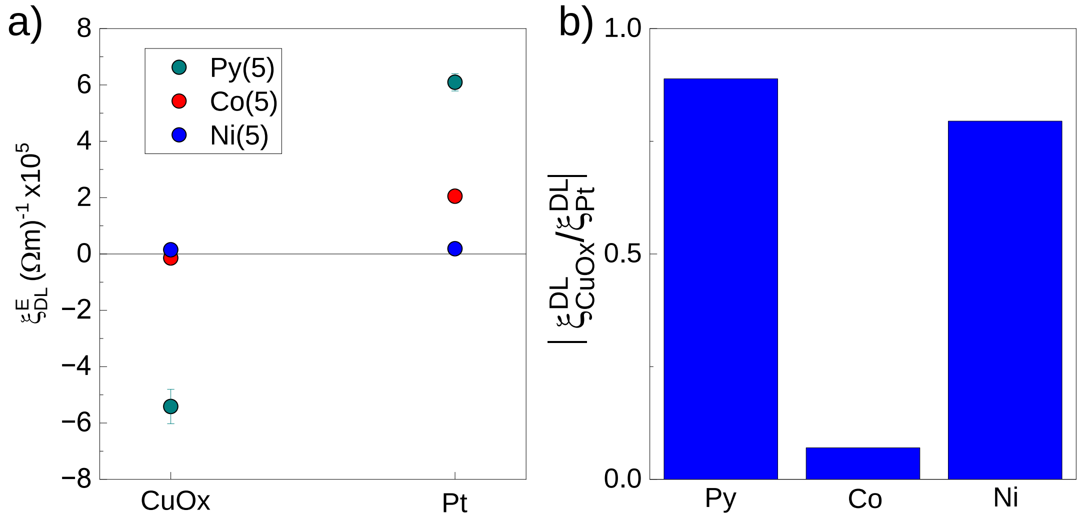

Figure S9 Spin transparency of the FM/NM interface. a) Plot of the damping-like torque efficiency (ξ_{DL}) in FM/CuO_X and FM/Pt bilayers with FM=Py (green), FM=Co (red) and FM=Ni (blue). b) Plot of the absolute value of the ratio between ξ_{DL} measured in each FM/CuO_X and corresponding FM/Pt reference.

8. Voltage-driven ion migration in Cu

Figure S10c displays the current vs. voltage (*I*-*V*) characteristic of a prototypical device under gating. As expected for an insulating barrier, the current is nearly zero at low gate voltages and becomes highly non-linear above $V_G \approx 3.5$ V, indicating non-ohmic resistance and no apparent leakage across the 25 nm thick GdO_X. Based on this curve, we choose $V_G \approx 3$V as the standard value for our experiments.

An indirect proof of the formation of metallic Cu via ion-migration upon gating with a positive gate voltage (V_G) comes from following the resistance (R_L) of the stack during the gating experiment. Upon applying a negative V_G, the Cu present in the stack gets oxidized and the resistance increases (light blue in Figure S10b). When V_G is set to zero after negative bias R_L remains stable, indicating that no further oxidation occurs. This is logical, as CuO_X and GdO_X are chemically stable at room temperature. On the other hand, upon applying a positive V_G (light red in Figure S10b), the O^{2-} ions migrate towards the top contact and out of CuO_X. Here the reduction process is not only limited to CuO_X but part of GdO_X can also loose oxygen. In this case, when gating is stopped after application of a positive V_G, R_L shows an initial sudden upturn (gray arrow in Figure S10b). This is due to the spontaneous re-oxidation of Gd that has a large affinity to oxygen. However, after this initial steep increase R_L, it tends to stabilize at a value of resistance close to the minimum reached after gating. This indicates that the re-oxidation of Cu to CuO_X is a much slower process. A similar behavior is also displayed by the device in Figure 4d of the

manuscript. To further test the stability of the formation of metallic Cu we have monitored the evolution of the sample resistance with time for over 15 h after gating. As it can be seen in Figure S10c, after the initial increase due to the oxidation of Gd, R_L increases slowly and never reaches its initial value. Therefore, the reduction of CuO_X to Cu is stable within the time frame of out OT measurements (which only takes minutes) and therefore, we believe that voltage-driven ion migration is effective for comparing the OT generated in metallic Cu and CuO_X. Indeed, we cannot estimate which portion of the thickness of the CuO_X layer gets reduced to metallic Cu, but the sign change of the torque indicates which species (Cu o CuO_X) predominates.

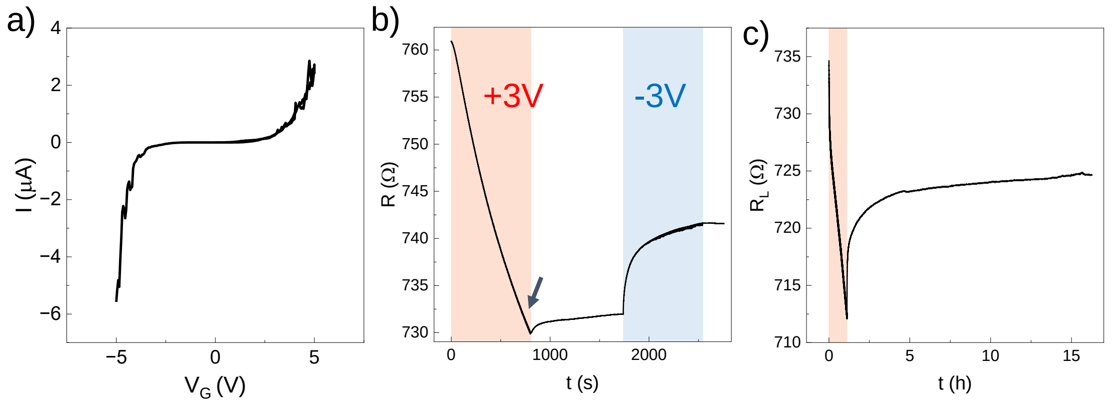

Figure S10 Evolution of R_L with gating. a) Plot of current (I) as a function of gate voltage (V_G) measured in 2-point geometry between -5 and 5 V. b) Plot of the longitudinal resistance (R_L) as a function of time (t) measured in two-pint geometry during a gating experiment. First a positive gate voltage (V_G=+3V red) is applied for 800s, then RL is left to stabilize for 1000s before a negative V_G=-3V (blue) is applied for 800s. The gray arrow indicates the initial steep increase of R_L after positive gating due to the re-oxidation of Gd. c) Plot of R_L as a function of t measured during more than 15 h after gating.

The reversible reduction of CuO_X to Cu is also reflected in the anomalous Hall effect (AHE) measurements shown in Figure S11a, which shows the plot of the Hall resistance as a function of out-of-plane field measured in the 3 stages of the gating experiment correspondent to Figure 5b of the main text. Initially, in the pristine state the 4-point resistance (R_{4w}) of the device was 83.6 Ω and R_{AHE}=0.45 Ω. After application of +3V during 2000s the Cu layer gets deoxidized after positive gating (R_{4w}=58.5 Ω)., and R_{AHE} is reduced to 0.2 Ω. This is because, at equal total applied current I, the current density in the magnetic Co layer is lower when Cu is metallic. Similarly, when Cu gets partly oxidized after 1000s of gating at -3V (R_{4w}=68.3 Ω), R_{AHE} rises back to 0.3 Ω as the current density in the magnetic layer increases.

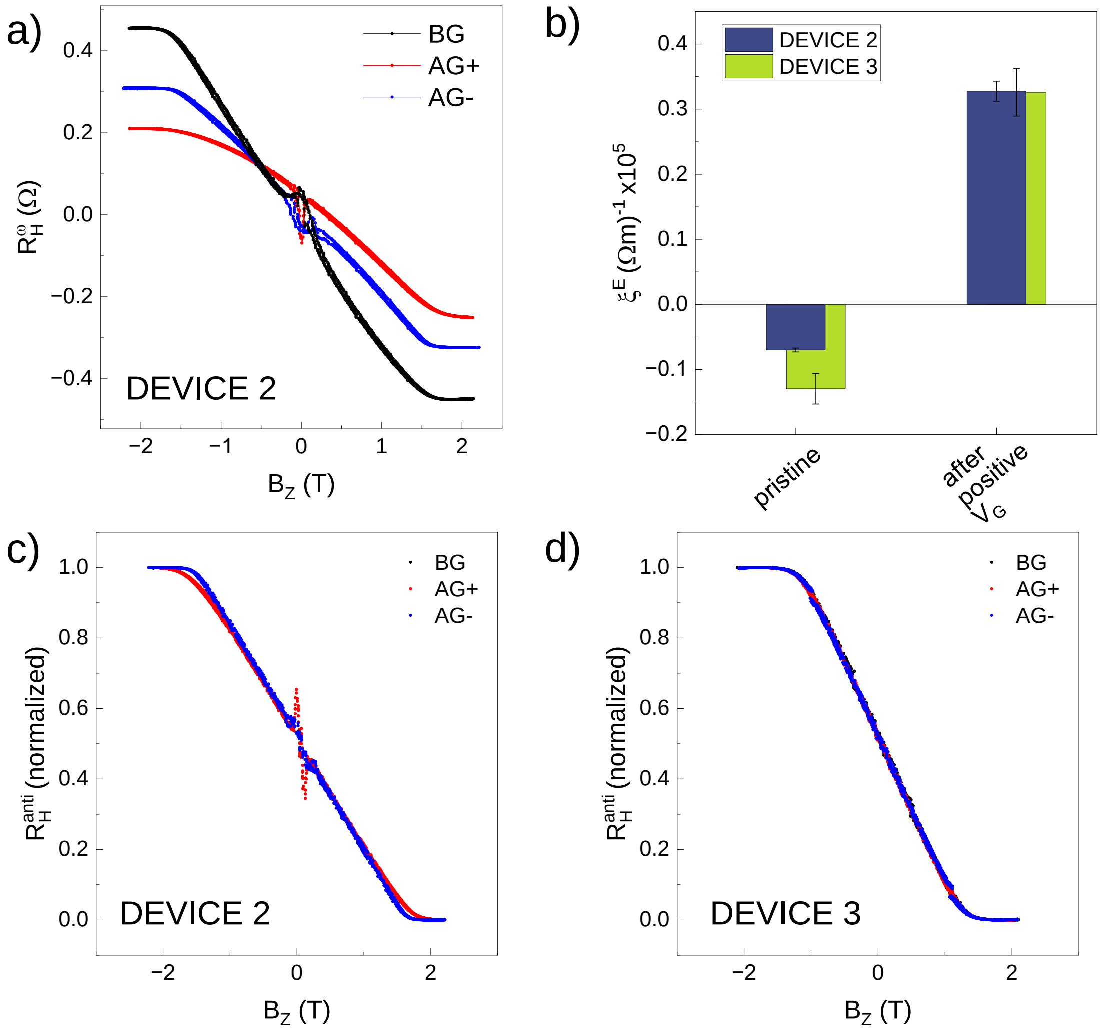

Figure S11 Evolution of the AHE with gating. a) Plot of the Hall resistance (R_H) as a function of out-of-plane field (B_z) of a Co/CuO_X gated device measured before gating (BG black), after application of a positive gate (AG+ red) and after application of a negative gate (AG− blue). b) Plot of the damping-like torque efficiency (ξ_{DL}) measured in two different devices in the pristine state and after gating with a positive gate voltage (V_G). c,d) Plot of the normalized antisymmetric component of the Hall resistance ($R^{anti}{}_{H}$) as a function of *Bz* of the two devices measured before gating (BG black), after application of a positive gate (AG+ red) and after application of a negative gate (AG− blue).

The measurement of the AHE also provides information on the oxidation state of the FM. Specifically the out-of-plane saturation field (H_S) is proportional to M_S as: $H_S = 2K_{eff}/\mu_0 M_S$, where K_{eff} is the anisotropy constant that combines contributions from shape anisotropy, crystalline anisotropy, and interface effects and μ_0 is the permeability of free space. H_S can be determined by measurements of the Anomalous Hall Effect (AHE), as shown in Figure 1b of the main text. In all our samples H_S is independent of the capping layer, but only on the thickness of the magnetic layer. To identify if minute changes in the saturation field (H_S) are present which could indicate a change of saturation magnetization due to partial oxidation of

Co we also calculated the antisymmetric part of the anomalous Hall resistance of Co before and after gating. The results from two prototypical devices are shown in Figure S11c-d. As it can be seen, in the case of device 2 (same shown in the main text) a small change of the shape of the AHE can be observed after application of a positive gate voltage (V_G). The original H_S is recovered after application of a negative gate voltage. On the other hand, for Device 3 no change is observed across the full gating cycle. Therefore, we conclude that oxidation of Co close to the Cu interface is possible and could occur in our experiments depending on the specific gating conditions. Note that this change of the shape of the AHE could also be caused by a change of interface anisotropy going from a Co/CuO_X to a Co/Cu interface. However, because the torque magnitude and sign measured for the two devices are the same within the error (Figure S11b), we conclude that the presence of CoO_X, if any, cannot be responsible for the large amplitude and sign variation that we observe when gating, which is instead due to the oxidation of Cu. Previous literature reports [*J. Feng et al., Phys. Rev. Applied 13, 044029 (2020); S. Wu et al., Appl. Phys. Lett. 122, 122403 (2023)*] have also shown that the presence of CoO_X has a rectification effect, enhancing the torque but not changing its sign. This is consistent with what measured for our reference samples where Co and Py were oxidized on purpose before the deposition of the Cu layer (see Note 5).
